# Ubiquitous arbuscular mycorrhizal fungi in the roots of herbaceous understory plants with hyphal degeneration in Colchicaceae and Gentianaceae

**DOI:** 10.1007/s00572-024-01145-9

**Published:** 2024-04-17

**Authors:** Ryota Kusakabe, Moe Sasuga, Masahide Yamato

**Affiliations:** 1 Graduate School of Horticulture, Chiba University, 648, Matsudo, Chiba, Matsudo 271-8510 Japan; 2 Graduate School of Education, Chiba University, 1-33, Yayoi-cho, Inage-ku, Chiba, 263-8522 Japan; 3 Faculty of Education, Chiba University, 1-33, Yayoi-cho, Inage-ku, Chiba, 263-8522 Japan

**Keywords:** AMF, Glomeromycotina, Hyphal degeneration, Partial mycohetereotrophy, 18S rDNA, Understory plants

## Abstract

Due to the loss of photosynthetic ability during evolution, some plant species rely on mycorrhizal fungi for their carbon source, and this nutritional strategy is known as mycoheterotrophy. Mycoheterotrophic plants forming *Paris*-type arbuscular mycorrhizas (AM) exhibit two distinctive mycorrhizal features: degeneration of fungal materials and specialization towards particular fungal lineages. To explore the possibility that some understory AM plants show partial mycoheterotrophy, i.e., both photosynthetic and mycoheterotrophic nutritional strategies, we investigated 13 green herbaceous plant species collected from five Japanese temperate forests. Following microscopic observation, degenerated hyphal coils were observed in four species: two Colchicaceae species, *Disporum sessile* and *Disporum smilacinum*, and two Gentianaceae species, *Gentiana scabra* and *Swertia japonica*. Through amplicon sequencing, however, we found that all examined plant species exhibited no specificity toward AM fungi. Several AM fungi were consistently found across most sites and all plant species studied. Because previous studies reported the detection of these AM fungi from various tree species in Japanese temperate forests, our findings suggest the presence of ubiquitous AM fungi in forest ecosystems. If the understory plants showing fungal degeneration exhibit partial mycoheterotrophy, they may obtain carbon compounds indirectly from a wide range of surrounding plants utilizing such ubiquitous AM fungi.

**Supplementary Information:**

The online version contains supplementary material available at 10.1007/s00572-024-01145-9.

## Introduction

The majority of terrestrial plants form arbuscular mycorrhizas (AM) with fungi of the subphylum Glomeromycotina (Spatafora et al. 2016; Brundrett and Tedersoo 2018). There are two main morphologies of AM: the *Arum*-type, which is characterized by intercellular hyphae and intracellular arbuscules, and the *Paris*-type, which is characterized by intracellular coiled hyphae that are often accompanied by intercalary arbuscules (arbusculate coils) (Gallaud 1905). The type of AM is primarily determined by the classification of host plant, especially at the family level (Smith and Smith 1997; Dickson et al. 2007), although in some cases, it can be determined by which AM fungi have colonized the plant (Cavagnaro et al. 2001; Dickson 2004). It is also confirmed that the number of plant families forming *Arum*-type AM and those forming *Paris*-type AM are similar (Dickson et al. 2007). Both types of AM enhance host plant growth and share expression patterns of AM-related genes in roots (Tominaga et al. 2022). However, their functional differences remain poorly understood.

Some plants have lost their photosynthetic capacity entirely and therefore fully rely on mycorrhizal fungi as their carbon source; this nutritional strategy is known as mycoheterotrophy (Leake 1994). Among mycoheterotrophic plants, Orchidaceae and Ericaceae plants have been found to associate with Basidiomycota and Ascomycota, whereas other plant families associate with Glomeromycotina (i.e., AM fungi). There are at least 330 species of mycoheterotrophic AM plants in nine angiosperm families (Merckx et al. 2013b; Cheek et al. 2024; Imhof 2024), and *Paris*-type AM have been observed in all plants examined (Imhof et al. 2013). Recently, Giesemann et al. (2021) suggested that partial mycoheterotrophy (mixotrophy), obtaining carbon compounds via both photosynthesis and mycoheterotrophy, may be common among chlorophyllous plants with *Paris*-type AM based on the enrichment of ^13^C and ^15^N. Along with the observation that understory plants typically develop *Paris*-type AM (Brundrett and Kendrick 1990; Yamato and Iwasaki 2002), it has been suggested that *Paris*-type AM may be a prerequisite for the evolution of mycoheterotrophy (Imhof 1999; Giesemann et al. 2021).

In general, mycoheterotrophic AM plants exhibit two distinct mycorrhizal characteristics. First, coiled intracellular hyphae develop in their roots and later undergo degeneration, which is thought to be caused by the digestion of the fungus by the host plant (Imhof et al. 2013). Degenerated hyphal coils also have been observed in (partial-) mycoheterotrophic plants within the Orchidaceae and Ericaceae. Fungal digestion is the mechanism by which mycoheterotrophic plants are hypothesized to obtain carbon compounds. Indeed, it has been confirmed that carbon is transferred to host plant cells from both living and degenerated fungal hyphal coils, with a larger quantity observed from the latter, in the protocorms of the photosynthetic orchid, *Spiranthes sinensis* (Kuga et al. 2014). In AM-forming green plants, fungal degeneration also has been confirmed in some species (e.g., Rath et al. 2013; Yamato et al. 2021). Second, AM-forming mycoheterotrophs exhibit specialization toward specific fungal lineages, predominantly the Glomeraceae (e.g. Merckx et al. 2012; Ogura-Tsujita et al. 2013; Yamato et al. 2014; Gomes et al. 2017), although the degree of specificity differs among the plant species (Merckx et al. 2012). However, it remains unclear whether *Paris*-type AM-forming green plants, which may be partial mycoheterotrophs, also exhibit specificity toward fungi.

Unlike plants in the Orchidaceae and Ericaceae, it can be difficult to identify partial mycoheterotrophy in AM-forming plants by examining their ^13^C and ^15^N abundances, because the stable isotopic signatures of AM fungi closely resemble those of host plants (Nakano et al. 1999; Merckx et al. 2010; Courty et al. 2011). For example, while Giesemann et al. (2021) found ^13^C enrichment in various *Paris*-type AM green plants thriving on shady forest grounds, Murata-Kato et al. (2022) reported that ^13^C enrichment was found not only in certain *Paris*-type AM plants but also in understory *Arum*-type AM and in nonmycorrhizal plants. Thus, ^13^C enrichment in understory plants can be affected by many factors other than mycoheterotrophy. In this study, we used fungal degeneration and fungal specificity rather than stable isotope signatures to explore the possibility of partial mycoheterotrophy in *Paris*-type AM green herbaceous plants. To do so, we conducted microscopic observations and amplicon sequencing of understory plant species collected from temperate forests in Japan.

## Materials and methods

### Study sites and sampling methods

Study sites were selected from five forests in the Kanto region of Japan. Sampling of understory herbaceous plants was conducted from May–October 2022 and May 2023 as follows: Kamogawa, Chiba Prefecture (in May, August, and October 2022), Tsukuba, Ibaraki Prefecture (July 2022), Funato, Chiba Prefecture (May 2022), Funyu, Tochigi Prefecture (May 2022), and Konbukuro, Chiba Prefecture (May 2023) (Fig. S1). Funato (alt. 20 m) and Konbukuro (alt. 20 m) are secondary forests with *Carpinus tschonoskii*, *Quercus serrata*, *Q. glauca*, etc. on alluvial soils. Kamogawa (alt. 275–325 m) is a natural mixed conifer-hardwood forest with *Abies firma*, *Tsuga sieboldii*, *Quercus* spp., *Castanopsis sieboldii*, *Machilus thunbergii*, etc. on brown forest soils. Funyu (alt. 290–355 m) is a *Chamaecyparis obtusa* plantation on Andosols. Tsukuba (alt. 25 m) is a planted broadleaf forest with *Quercus* spp., *Celtis sinensis*, *Fagus crenata*, *Acer* spp., etc. on pale Andosol soils. At each study site, we collected two to five individuals of each plant species for which conspecific or closely related species had been reported to form *Paris*-type AM in previous studies (summarized by Dickson et al. 2007). Conspecific plant samples were at a distance of one to several hundred meters from one another, depending on the species. In total, 61 individuals of 13 species representing 9 families were collected, with some species obtained at more than one study site. Plant collection details are shown in Table S1. After sampling, roots were washed in tap water and all fine roots (unlignified roots less than 2 mm in diameter) were collected. The fine roots were randomly divided into two subsamples per individual, one of which was used for molecular analysis and the other for morphological observation. All root samples were stored in 99.5% and 70% ethanol solutions for further molecular and morphological analyses, respectively.

### Morphological observation

Fine root subsamples retrieved from individual plants were first autoclaved at 121℃ for 20 min in 10% KOH. Root samples were then stained using 0.05% trypan blue in lactic acid at 90℃ for 10 min or 0.1% chlorozol black E in lactic acid at 99℃ for 2 h. Stained roots were squashed using a cover glass. Stained root slides were observed under a BX51 microscope equipped with differential interference contrast optics (Olympus, Tokyo, Japan) and were photographed with a DP72 CCD camera (Olympus). Total and degenerated AM fungal colonization rates of each individual were analyzed by use of the magnified intersection method (McGonigle et al. 1990) with approximately 100 intersections for approximately 10 cm of root length per individual.

### Molecular analysis

For molecular analysis, we first extracted total DNA from the fine root subsamples of each plant individual using the DNeasy Plant Mini Kit (Qiagen, Hilden, Germany). DNA extracts were then quantified using the QuantiFluor® dsDNA System and a Quantus™ Fluorometer (Promega, Tokyo, Japan). DNA extracts with high concentrations were diluted with TE buffer to 5 ng µL^− 1^. For the first PCR amplification, the partial nuclear small subunit ribosomal RNA gene (SSU rDNA), approximately 260 bp in length including primers, was amplified from extracted DNA using the AM fungal-specific primers AMV4.5NF and AMDGR (Sato et al. 2005). This primer set favors amplification of Glomeraceae sequences at the expense of Ambisporaceae, Claroideoglomeraceae, and Paraglomeraceae sequences (Van Geel et al. 2014). Nextera Transposase Adapter Reads 1 and 2 were linked to the 5′-ends of AMV4.5NF and AMDGR, respectively, for sequencing using an Illumina Nextera Library Prep Kit (Illumina, San Diego, CA, USA). The mixture of the first PCR contained 1 µL DNA extract (up to 5 ng µL^− 1^), 12.5 µL of 2× KAPA HiFi Hot Start Ready Mix (Kapa Biosystems, Woburn, MA, USA), and 0.3 µM of each primer in a total volume of 25 µL. The PCR program was performed on a Thermal Cycler Gene Atlas (ASTEC, Fukuoka, Japan) as follows: initial denaturation at 95℃ for 3 min, followed by 35 cycles at 98℃ for 20 s, 60℃ for 15 s, and 72℃ for 15 s before a final elongation at 72℃ for 5 min. The PCR products were purified using AMpure XP (Beckman Coulter, Brea, CA, USA). The purified PCR products were quantified as described above, before diluting them with TE buffer to 5 ng µl^− 1^ for use as templates in a second PCR. The second PCR reaction mixture contained 2 µL of the first PCR product, 6 µL of 2× KAPA HiFi Hot Start Ready Mix, and 2 µL of each Index Primer of the Nextera XT Index Kit (Illumina) in a total volume of 12 µL. The second PCR was conducted using the following program: 95℃ for 3 min, followed by 12 cycles at 98℃ for 10 s, 55℃ for 30 s, and 72℃ for 30 s. The products of the second PCR were purified using AMpure XP and then pooled in equimolar quantities to prepare a sequence library. This library was sequenced using an Illumina NovaSeq 6000 with 2 × 250 bp paired-end reads, and all sequencing was performed by a commercial sequencing service (Rhelixa, Tokyo, Japan). Sequence data were generated in FASTQ format for each index primer pair and deposited in the DDBJ sequence archive (DRA) under accession number PRJDB17537.

### Bioinformatics

After sequencing, demultiplexed sequences were processed using QIIME 2 version 2023.7 (Bolyen et al. 2019) as follows. First, amplicon sequence variants (ASVs) were generated from raw paired-end sequences using the DADA2 plugin (Callahan et al. 2016). The first 20 and 22 nucleotides at the 5′-end of both the forward and reverse reads, respectively, were trimmed. The 3′-end of both reads was then truncated at position 145. Taxonomic classification of ASVs was conducted using the pretrained SILVA 138 classifier (Quast et al. 2013; Robeson et al. 2021) and employing classify-sklearn in the feature-classifier plugin (Bokulich et al. 2018). After removing non-AM fungal ASVs, the remaining ASVs were clustered into operational taxonomic units (OTUs) by 97% similarity threshold using the cluster-features-de-novo command of the VSEARCH plugin (Rognes et al. 2016). After removing rare OTUs (i.e., < 10 reads), the OTU composition data set was imported to R version 4.3.1. To avoid bias caused by different numbers of reads for different samples, all reads were rarefied to 4665 per sample using the “rrarefy” function of the vegan package version 2.6–4 (Oksanen et al. 2022). A rarefaction curve was then plotted for each plant individual to confirm efficient sampling; this was performed using the “ggrare” function implemented in the ranacapa package version 0.1.0 (Kandlikar et al. 2018) (Fig. S2). After rarefaction analysis, the remaining OTUs were sequentially numbered on the basis of the decreasing order of sample detection then used for subsequent analyses. A heatmap was plotted on the basis of the relative abundance of the top 20 OTUs with the highest number of detected plant individuals using the function “plot_heatmap” as implemented by the phyloseq package version 1.46.0 (McMurdie and Holmes 2013).

### Assignment to VTXs in Maarj*AM*

The Maarj*AM* database (Öpik et al. 2010; https://maarjam.ut.ee), which classifies AM fungal partial SSU rDNA sequences into virtual taxa (VTX) based on 97% similarity, was used to infer the distribution and potential host plants for each OTU acquired in this study. Representative OTU sequences were assigned to VTX-type sequences using the BLAST + algorithm (Camacho et al. 2009), specifically, for an identity of ≥ 97%, query coverage ≥ 80%, and BLAST e-value < 1e-50. However, for OTUs that could not be assigned using these criteria, we used the closest VTX-type sequences as determined by the following relaxed criteria, as specified by Suetsugu and Okada (2021): sequence similarity ≥ 90%, a BLAST e-value ≤ 1e-50, and query coverage ≥ 80%. If the relaxed criteria were met, each OTU was classified as the family of the corresponding VTX; otherwise, it was classified as the order. Claroideoglomeraceae was treated as Entrophosporaceae in the order Entrophosporales (Błaszkowski et al. 2022). Finally, we note that genus-level systematic analyses were not performed using Maarj*AM*, because they are not up-to-date.

### Phylogenetic analysis

To clarify the phylogenetic position of each OTU, we conducted a phylogenetic analysis. For the analysis, SSU rDNA sequences of described Glomeromycotina species were downloaded from the International Nucleotide Sequence Database (INSD). A multiple sequence alignment was performed for the representative sequences of each OTU and downloaded data using MAFFT version 7.520 (Katoh and Standley 2013). For the aligned data set composed of 255 nucleotides, maximum likelihood analysis with 1,000 bootstrap replicates (Felsenstein 1985) was then performed using RAxML-NG version 1.2.0 (Kozlov et al. 2019). The best fit model was selected according to the corrected Akaike information criterion (AICc; Sugiura 1978) by ModelTest-NG version 0.1.7 (Darriba et al. 2020). Next, a phylogenetic tree was drawn using the function “ggtree”, and a heatmap based on the presence/abundance data of each OTU in each plant species was plotted using the function “gheatmap”, both as implemented by the ggtree package version 3.10.0 in R (Yu et al. 2017).

## Results

### Morphological observations

**Fig. 1:**
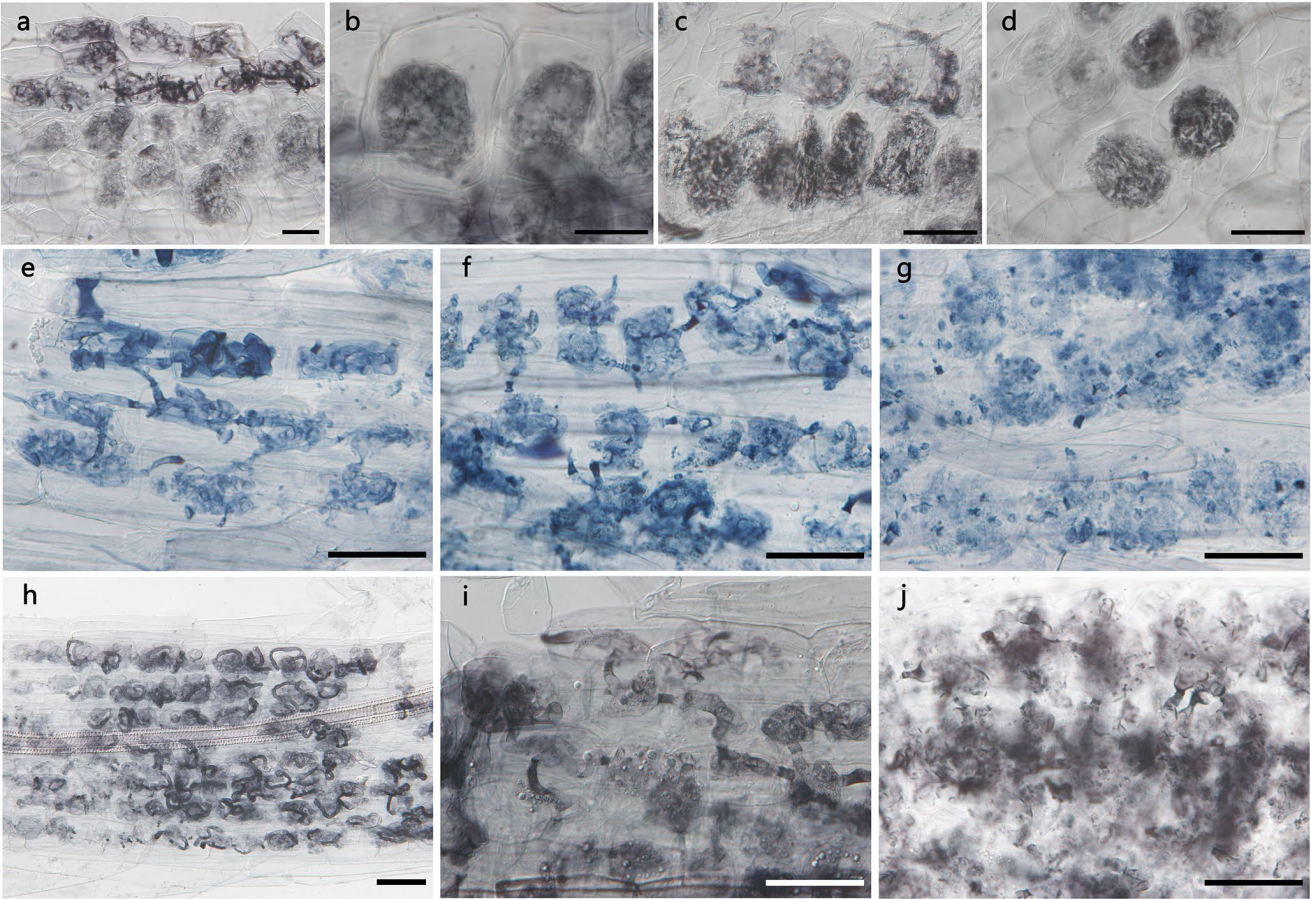
Mycorrhizal structures of *Disporum sessile* (**a, b**) and *Disporum smilacinum* (**c, d**) (Colchicaceae) stained with Chlolazol black E, *Gentiana scabra* stained with trypan blue (**e, f, g**) and *Swertia japonica* stained with Chlolazol black E (**h, i, j**) (Gentianaceae). **a**, **c** Hyphal coils (upper side) and degenerated hyphal coils (lower side). **b**, **d** Degenerated hyphal coils. **e**, **h** Hyphal coils. **f**, **i** Hyphal swellings and degenerated hyphae. **g**, **j** Indistinct fungal materials that seemed to have undergone further degeneration. Bars: 50 μm

All 13 plant species investigated in this study formed *Paris*-type AM, which were characterized by intracellular hyphal coils and cell-to-cell spread. Degenerated fungal materials were observed in four species: two Colchicaceae species, *Disporum sessile* and *Disporum smilacinum*, and two Gentianaceae species, *Gentiana scabra* and *Swertia japonica* (Fig. 1). These species, except for *Gen. scabra*, were collected multiple times throughout the year, while many other species were collected only once during the study period (e.g., *Asarum nipponicum*, *Chloranthus quadrifolius*, *Tricyrtis affinis*; Table S1). The total colonization and degeneration rates for each plant individual are shown in Table S1, and these values differed greatly among individuals.

In *D. sessile* and *D. smilacinum* (Colchicaceae), intact hyphal coils (Fig. 1a, c), arbusculate coils, and vesicles (Fig. S3) were observed along with hyphal coils that had degenerated to amorphous clumps (Fig. 1a–d). A total of fourteen *D. sessile* individuals were collected at four sites, in which degenerated fungal materials were observed in seven individuals at Tsukuba and Konbukuro. The sites where no degenerated fungal materials were observed in *D. sessile* were sampled in early May. For *D. smilacinum*, a total of six individuals were collected from Funyu in late May and Tukuba in early July, but fungal degeneration was observed only in three individuals from Tukuba.

The mycorrhizal structures of *Gen. scabra* and *S. japonica* (Gentianaceae) were similar, with intact hyphal coils (Fig. 1e, h) and arbusculate coils (Fig. S3). The hyphal coils often had irregular swellings, and some had degenerated into amorphous clumps in both species (Fig. 1f, i). In addition, we also observed indistinct fungal materials that appeared to have undergone further degeneration (Fig. 1g, j). Moreover, the cell-to-cell hyphae were darkly stained and remained dark even after fungus degeneration (Fig. 1e–j). Degenerated fungal materials were observed in all seven *S. japonica* samples from Tsukuba and Konbukuro. Excluding one exceptionally low value (0.9%), the ratio of degenerated fungal materials in root samples collected from Kamogawa in October (43.6% ± 3.89; mean ± SE) exceeded those collected from Tsukuba in July (14.7% ± 5.58). *Gen. scabra* was collected only from Kamogawa in August, and degenerated fungal materials were observed in one of the three samples.

### AM fungal community

Illumina NovaSeq 6000 sequencing yielded 3,798,397 raw reads. After processing with DADA2 and excluding non-AM fungal sequences, we obtained 2,779,072 reads. These were classified into 835 ASVs and 180 OTUs. Following the removal of rare OTUs (< 10 reads in total) and the execution of rarefaction analysis, 146 OTUs remained. These OTUs were numbered in decreasing order based on the number of detected samples and were used for subsequent analyses. The representative sequences of each OTU were then deposited in the INSD under the accession numbers TABA01000001–TABA01000146.

**Fig. 2:**
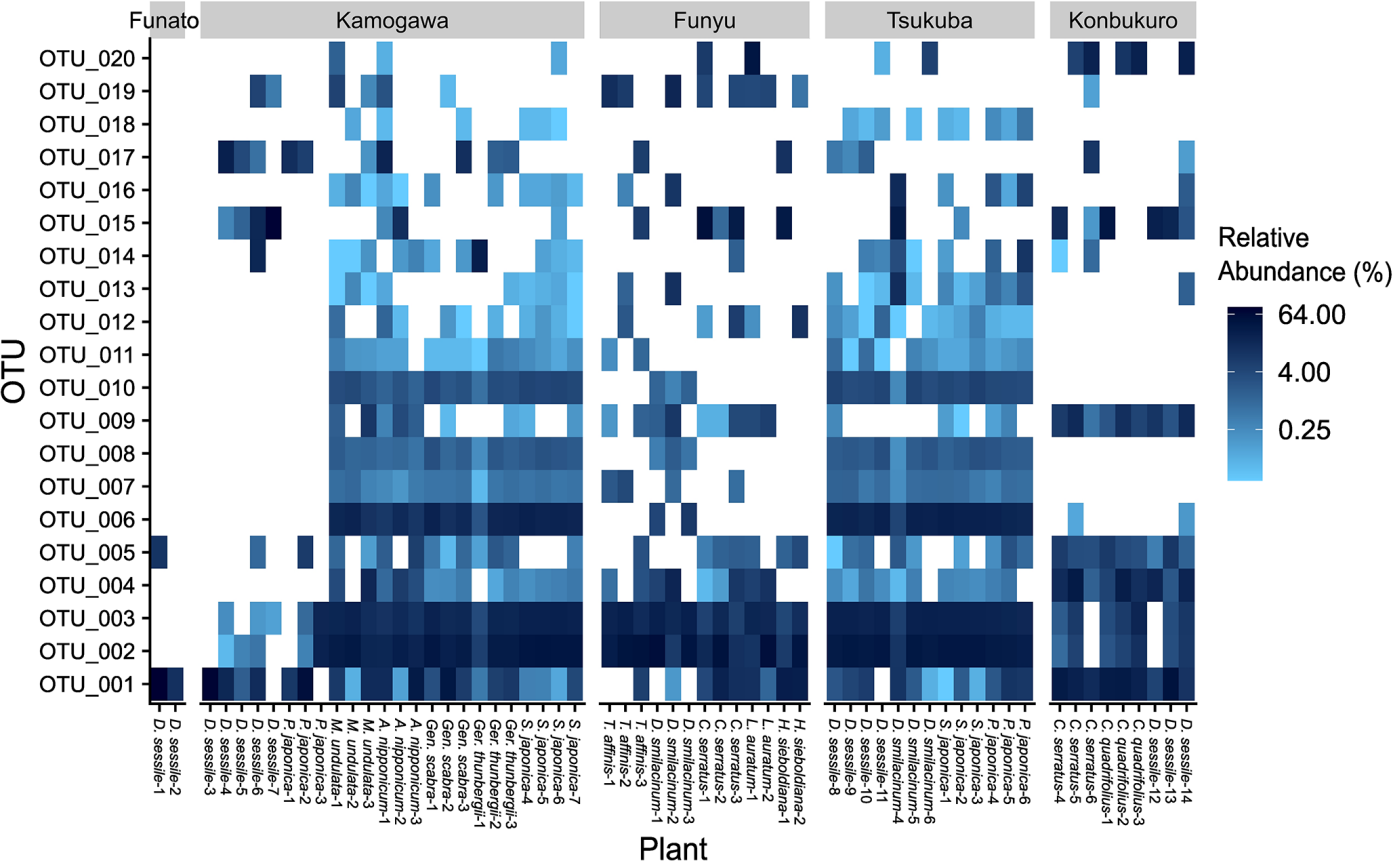
Heatmap of AM fungal OTUs based on relative abundances (%) estimated by read counts within each plant individual. Only the top 20 OTUs with the highest number of detected plant individuals are presented

The phylogenetic tree and presence/absence matrix of the AM fungal OTUs are shown in Fig. S4. The phylogenetic tree revealed that many OTUs were not closely related to known species. Moreover, the number of AM fungal OTUs detected in each plant individual ranged from 3 to 34 (Table S1, Fig. S5), and in each plant species from 9 to 71 (Fig. S4). Most plant species were associated with phylogenetically diverse AM fungi; i.e., those that belonged to two or more orders. Certain OTUs exhibited widespread distributions across study sites and plant species. For instance, out of the 61 plant individuals from 13 species at 5 sites examined, OTU_001 was detected in 54 individuals from all 13 species at 5 sites, and OTU_002 and OTU_003 were detected in 54 individuals from all 13 species at 4 sites (Fig. 2). Several OTUs, including these widely distributed ones, showed high relative abundances as estimated by read counts within each plant individual.

### Assignment to VTXs in Maarj*AM*

Next, BLAST searches of the 146 OTUs against the VTX-type sequences in Maarj*AM* (Table S2) revealed that 86 OTUs were assigned to the VTXs (≥ 97% identity). The three most ubiquitous OTUs (i.e., OTU_001, 002, and 003) corresponded to VTX 166, 84, and 80, respectively. The remaining OTUs, except for OTU_102, met the relaxed criteria (i.e., ≥ 90% identity). Several OTUs were found to be equally close to two VTXs; for instance, OTU_014 exhibited identical BLAST results for VTX113 and 387.

Based on the relaxed criteria, 145 OTUs were classified into eight families: Glomeraceae (106 OTUs), Acaulosporaceae (12 OTUs), Gigasporaceae (7 OTUs), Ambisporaceae (7 OTUs), Archaeosporaceae (5 OTUs), Entrophosporaceae (5 OTUs), Diversisporaceae (2 OTUs), and Paraglomeraceae (1 OTU). One remaining OTU, OTU_102, failed to meet the relaxed criteria and was classified on the order level (Diversisporales).

## Discussion

### Mycorrhizal structures

In this study, we examined the mycorrhizal structures of 13 understory plant species and found degenerated fungal materials in four of them, *D. sessile*, *D. smilacinum*, *Gen. scabra*, and *S. japonica*. The presence and ratio of degenerated fungal materials varied among the examined samples in all four species, and these variations may be related to local environmental factors such as light conditions. A negative correlation between light availability and the degree of dependence on fungal carbon sources has been reported for the partial mycoheterotrophic *Pyrola japonica* (Ericaceae; Matsuda et al. 2012). Alternatively, plant growth stages or seasons also may influence degeneration, because samples showing no degenerated fungal materials or low rates were collected earlier in the season.

To our knowledge, fungal degeneration in AM-forming green plants has been confirmed in some plant species to which fully or initially mycoheterotrophic plants belong (McGee 1985; Kovács et al. 2003; Rath et al. 2013; Sýkorová 2014; Yamato et al. 2021). In this study, however, degenerated fungal materials were observed in two Colchicaceae species, *D. sessile* and *D. smilacinum*, and this plant family has never been known to have fully mycoheterotrophic AM plants.

Notably, *Gen. scabra* and *S. japonica* examined in this study belong to the tribe Gentianeae, and the mycorrhizal structures of different Gentianeae species in previous studies are highly similar (Sýkorová 2014; Yamato et al. 2021). Furthermore, these mycorrhizal structures also resemble those of full mycoheterotrophic *Voyria aphylla* in the Voyrieae (Imhof 1999) as well as photosynthetic *Centaurium* spp. in the Chironieae (McGee 1985). The family Gentianaceae comprises seven tribes, and full mycoheterotrophy within the Gentianaceae has evolved independently at least four times, including in *Voyria* (Voyrieae), *Voyriella* (Saccifolieae), and *Exochaenium* and *Exacum* (Exaceae) (Merckx et al. 2013a). Therefore, it is plausible that pre-adaptation for mycoheterotrophy occurred during the family differentiation. Actually, partial mycoheterotrophy in the Gentianaceae has been suggested for some species within the tribe Gentianeae (Cameron and Bolin 2010; Suetsugu et al. 2020; Giesemann et al. 2021). However, some Gentianaceae species, including *Gen. scabra* and *S. japonica*, can be pot-cultured, so even if these plants have partial mycoheterotrophy, they may not be highly dependent on fungi.

The presence of fungal degeneration in these four species suggests a potential for partial mycoheterotrophy. Degeneration alone, however, does not demonstrate the supply of biologically meaningful amounts of fixed carbon compounds to a host plant. Therefore, further investigation is required to confirm partial mycoheterotrophy in these plants. In this study, many species were collected from a single site within a single day. Hence, the possibility of degeneration cannot be excluded for plant species in which degenerated fungal materials were not confirmed in this study.

### AM fungal specificity

Most plant species investigated in this study had phylogenetically diverse AM fungi that belonged to two or more orders. In the case of *L. auratum*, nine OTUs within the family Glomeraceae were detected, yet these OTUs belonged to various lineages within the family. Based on the presence/absence of the OTU matrix, we concluded that all plant species examined, including those with degenerated fungal materials, showed no specificity toward AM fungi.

### AM fungal community

The phylogenetic tree revealed that many OTUs were not closely related to sequences from known AM fungal species. Currently, 346 AM fungal species have been described (http://www.amf-phylogeny.com/amphylo_species.html), but SSU rDNA sequences have been obtained for only a fraction of them (Öpik et al. 2014). Furthermore, molecular analyses of environmental samples have estimated the presence of numerous undescribed AM fungi (Kivlin et al. 2011). To date, most of the AM fungi that have been described have originated from human-impacted habitats, and a relatively high number of undescribed species are anticipated in forest ecosystems (Hart et al. 2014).

The OTUs 001, 002, and 003 were consistently found in most plant individuals, and they correspond to the VTX166, 84, and 80 in Maarj*AM*, respectively. Furthermore, VTX166 has been identified as *Dominikia aurea* (Glomeraceae) or a closely related species according to Kusakabe and Yamato (2023). These VTXs have been widely detected in tree species growing in temperate Japanese forests (Miyake et al. 2020; Matsuda et al. 2021; Djotan et al. 2023). These VTXs also have been detected in phylogenetically independent fully mycoheterotrophic plants (Öpik et al. 2010; https://maarjam.ut.ee); thus, they are likely to be cheating susceptible fungi, as suggested by Perez-Lamarque et al. (2020). If the understory plants showing fungal degeneration exhibit partial mycoheterotrophy, they may obtain carbon compounds indirectly from a wide range of surrounding plants by utilizing such ubiquitous AM fungi. A previous study also suggested that fully mycoheterotrophic plants preferentially target AM fungi that are well connected to surrounding autotrophic plants (Gomes et al. 2022).

## Conclusions

We conducted microscopic observations and amplicon sequencing on the roots of 13 understory herbaceous photosynthetic plant species that form *Paris*-type AM. The results showed that degenerated fungal materials were observed in four of the examined plant species, and all species examined did not exhibit specificity for particular fungal lineages. The fungal degeneration suggests the possibility of acquiring carbon compounds from mycorrhizal fungi, i.e., partial mycoheterotrophy, although the fungal degeneration alone does not provide direct evidence of the supply of carbon compounds to the host plant. Hence, further study is necessary to validate partial mycoheterotrophy in these plants. Additionally, our data suggest the presence of ubiquitous AM fungi in forest ecosystems. If the understory plants showing fungal degeneration exhibit partial mycoheterotrophy, they may utilize a wide range of surrounding plants as carbon sources by targeting the ubiquitous AM fungi.

### Electronic supplementary material

Below is the link to the electronic supplementary material.

Supplementary Material 1

Supplementary Material 2

Supplementary Material 3

## Data Availability

Sequence data were generated in FASTQ format for each index primer pair and deposited in the DDBJ sequence archive (DRA) under accession number PRJDB17537.The representative sequences of each OTU were then deposited in the INSD under the accession numbers TABA01000001–TABA01000146.
