# Supplementary material for: Ubiquitous arbuscular mycorrhizal fungi in the roots of herbaceous understory plants with hyphal degeneration in Colchicaceae and Gentianaceae
Source: Mycorrhiza. 2024 Apr 17;34(3):181–90. doi: 10.1007/s00572-024-01145-9 (PMC11166799; doi:10.1007/s00572-024-01145-9)
Supplement: Supplementary file 1 — Supplementary Material 1 [file 572_2024_1145_MOESM1_ESM.pdf]

Ubiquitous arbuscular mycorrhizal fungi in the roots of herbaceous understory plants with hyphal degeneration in Colchicaceae and Gentianaceae plants

Ryota Kusakabe[1], Moe Sasuga[2], Masahide Yamato[3]

[1]Graduate School of Horticulture, Chiba University, 648, Matsudo, Matsudo, Chiba 271-8510, Japan

[2]Graduate School of Education, Chiba University, 1-33, Yayoi-cho, Inage-ku, Chiba 263-8522, Japan

[3]Faculty of Education, Chiba University, 1-33, Yayoi-cho, Inage-ku, Chiba 263-8522, Japan

Supplementary Figure

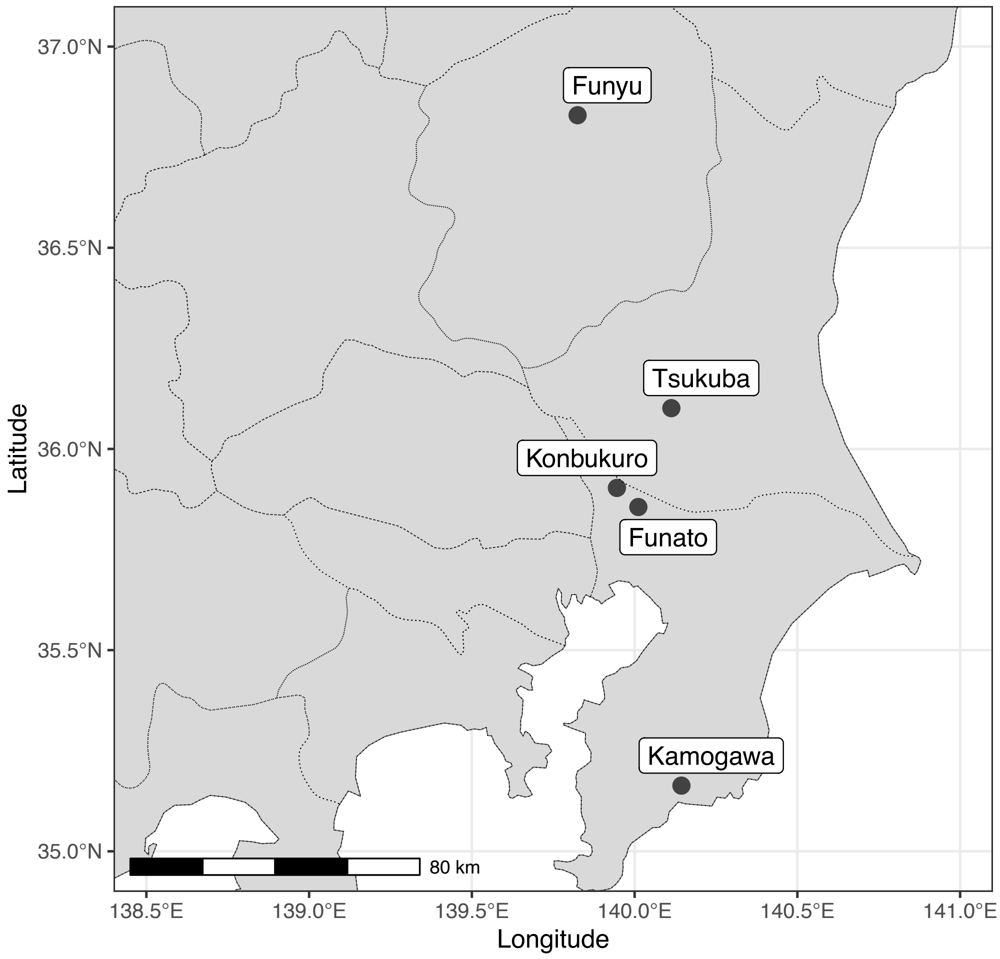

Fig. S1 Location of the study sites

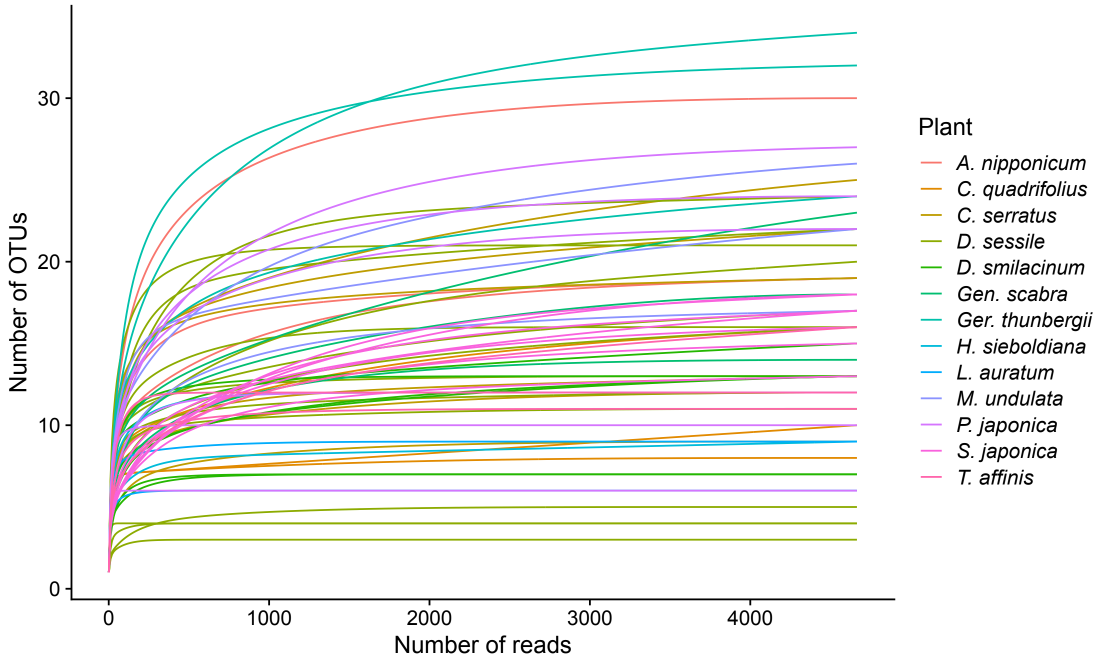

Fig. S2 Rarefaction curves of arbuscular mycorrhizal fungal OTUs against the number of reads after rarefication to 4665 reads per sample

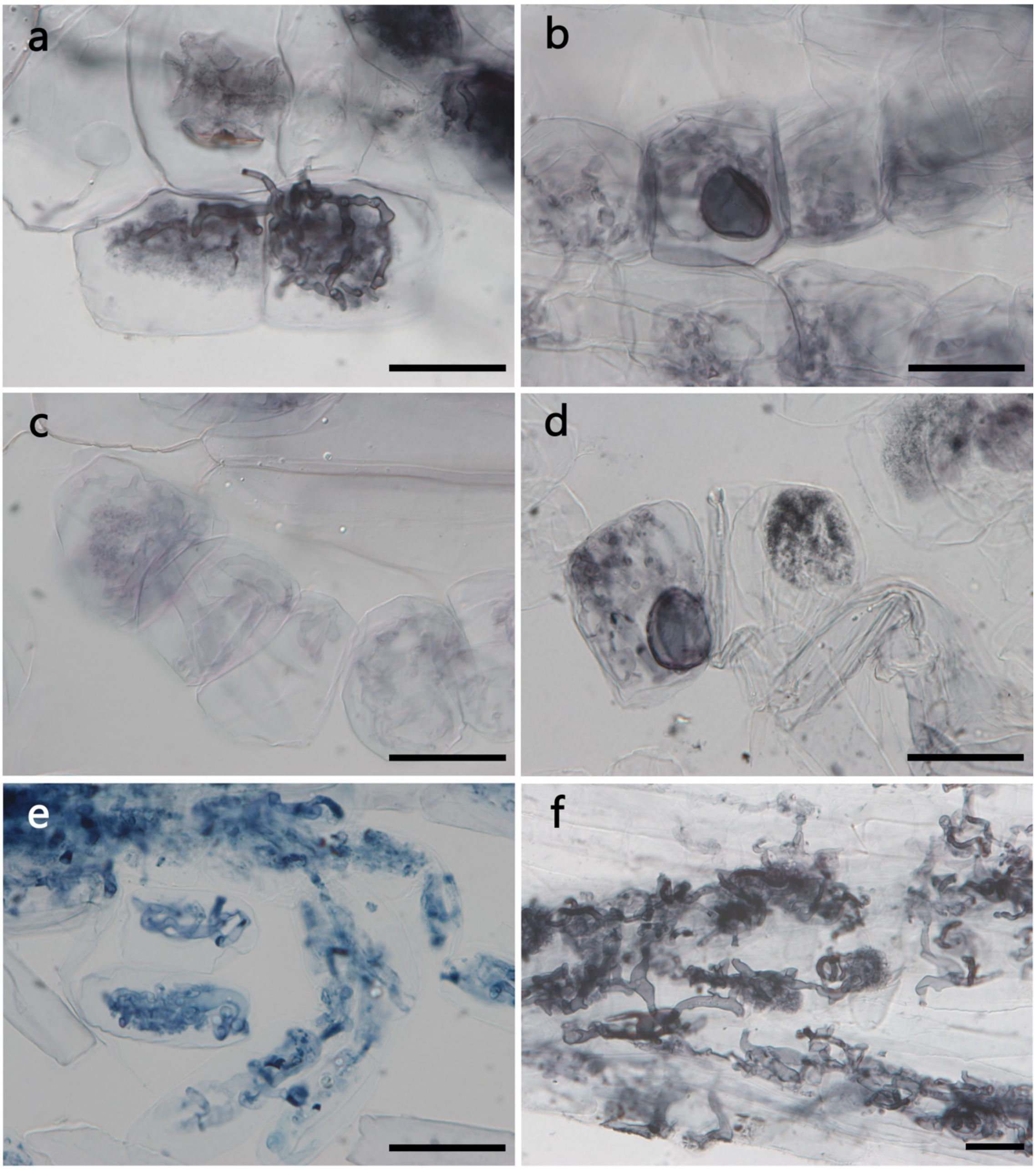

Fig. S3 Mycorrhizal structures of *Disporum sessile* (**a, b**), *Disporum smilacinum* (**c, d**), *Gentiana scabra* (**e**), and *Swertia japonica* (**f**). **a** Arbusculate coils and degenerated fungal material. **b** Vesicle. **c, e, f** Arbusculate coils. **d** Vesicle and degenerated fungal materials. Bars: 50 μm

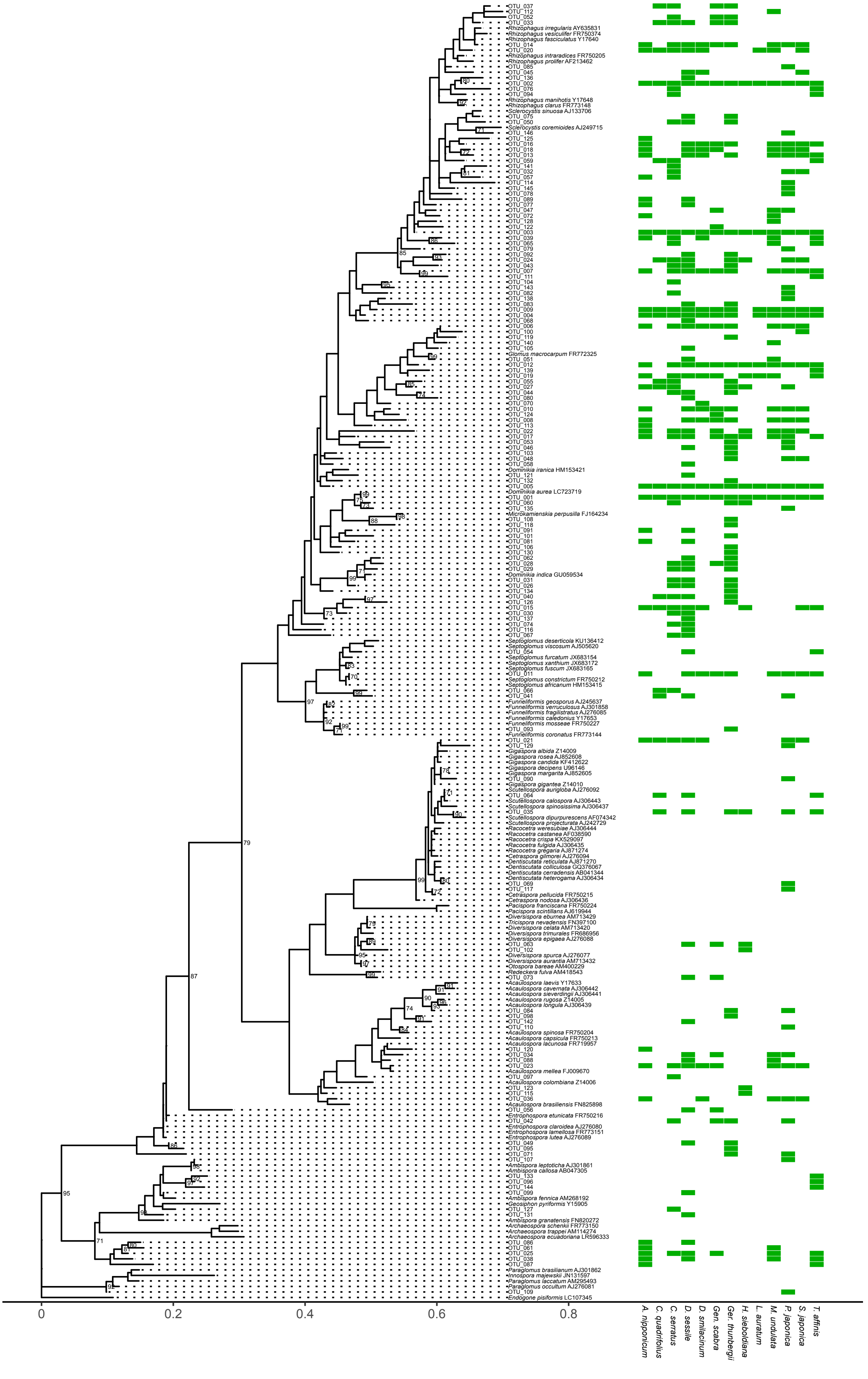

0
0.2
0.4
0.6
0.8
A. nipponicum
C. quadrifolius
C. serratus
D. sessile
D. smilacinum
Gen. scabra
Ger. thunbergii
H. sieboldiana
L. auratum
M. undulata
P. japonica
S. japonica
T. affinis

Fig. S4 Maximum likelihood phylogenetic tree based on partial SSU rDNA of arbuscular mycorrhizal fungi and presence/absence OTU matrix in each plant species. The tree consists of representative sequences from each OTU obtained in this study and known species downloaded from International Nucleotide Sequence Database (INSD). INSD accession numbers are given for downloaded sequences. The JC model is selected as the best-fit nucleotide substitution model based on the corrected Akaike information criterion (AICc). The tree is rooted with *Endogone pisiformis* (LC107345). Bootstrap values with 1000 replications are depicted at each node, showing only values >70%. The scale bar indicates the number of substitutions per site

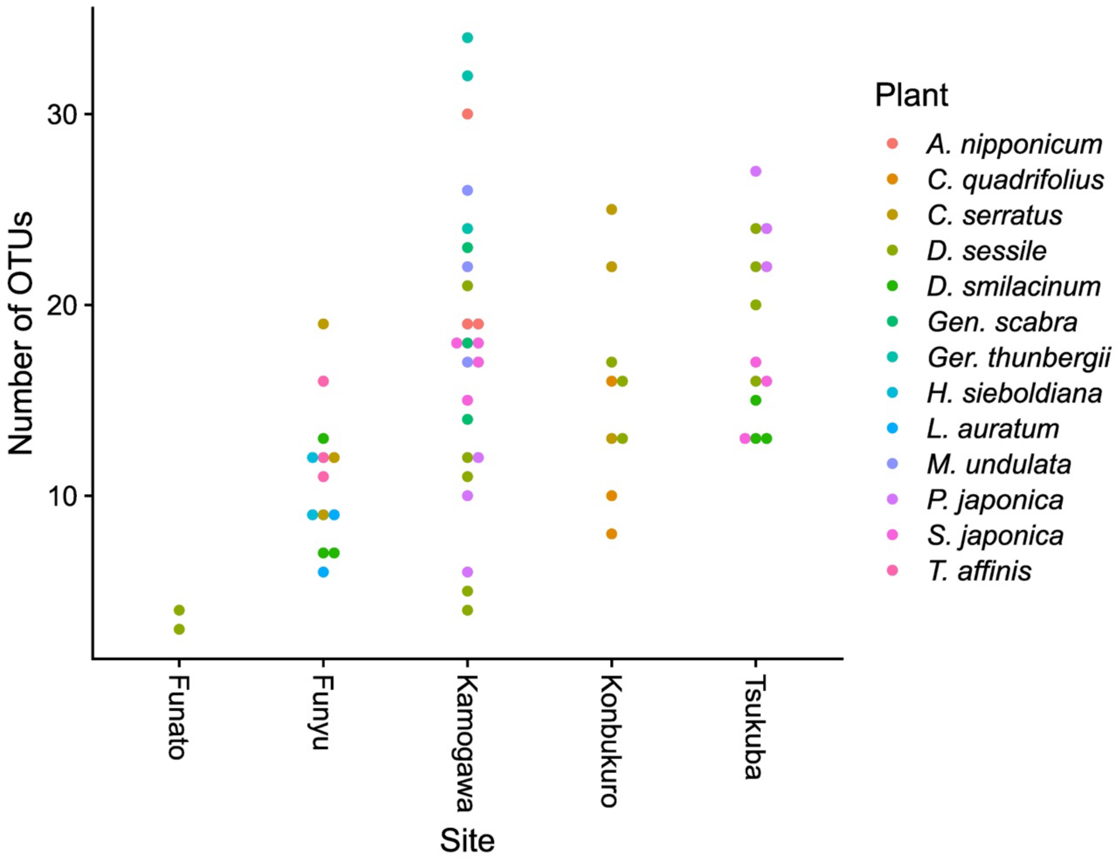

Fig. S5 Number of AM fungal OTUs in each plant individual
